# Supplementary material for: A putative ABC transporter gene, CcT1, is involved in beauvericin synthesis, conidiation, and oxidative stress resistance in Cordyceps chanhua
Source: Microbiol Spectr. 2025 May 22;13(7):e03425-24. doi: 10.1128/spectrum.03425-24 (PMC12210886; doi:10.1128/spectrum.03425-24)
Supplement: Supplemental material — Fig. S1; Tables S1 and S2. [file spectrum.03425-24-s0001.docx]

**SUPPLEMENTARY MATERIALS**


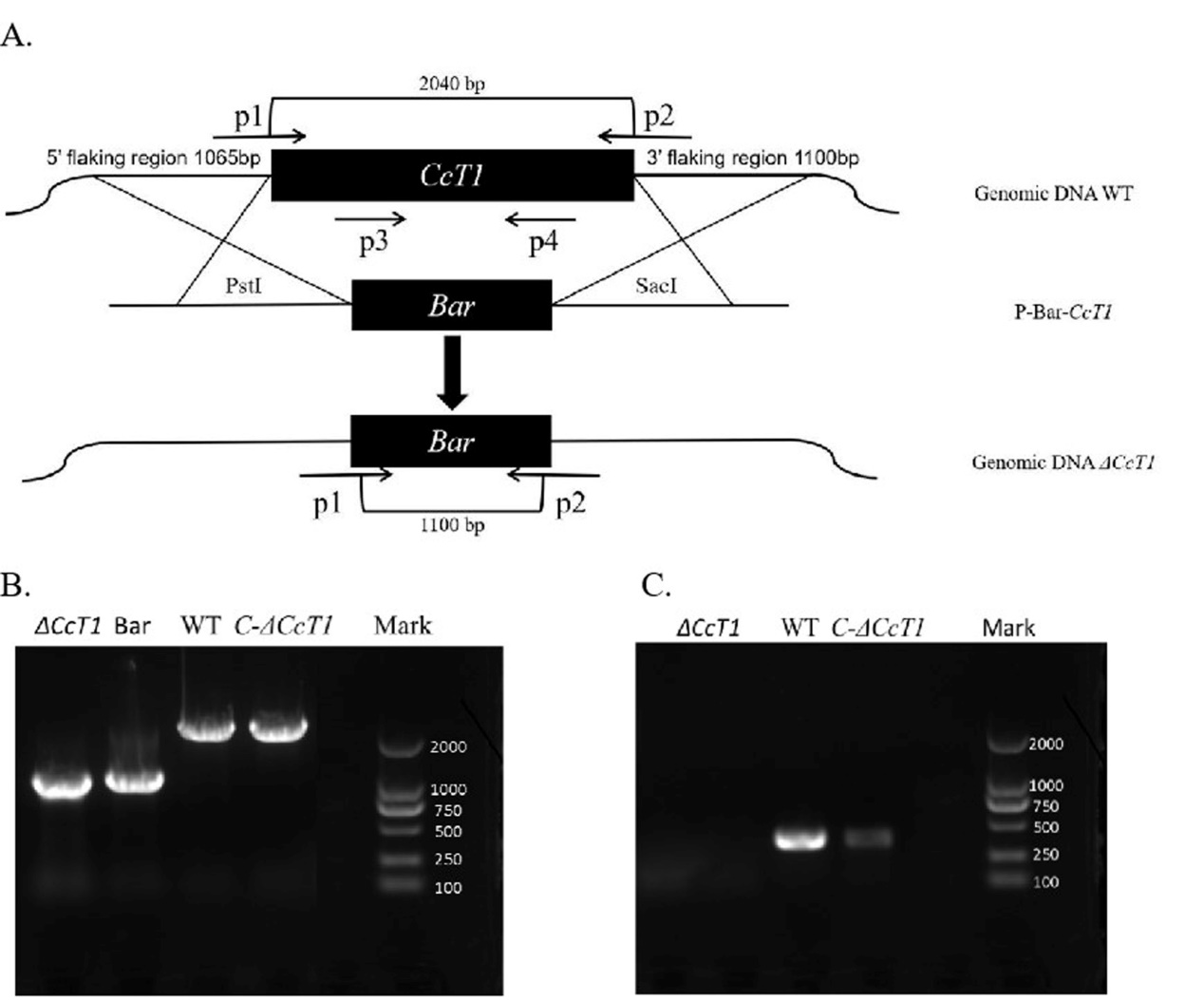


Fig. S1. Acquisition of Δ*CcT1* and C-Δ*CcT1* (A) Schematic diagram of *CcT1* gene disruption via homologous recombination. Within this diagram, the 5' and 3' segments of *CcT1*, (1065 and 1100 bp) along with the overlapping regions in the P-Bar-*CcT1* (recombinant vector), are employed for the precise replacement of a portion of *CcT1* with a 1100-base pair Bar plasmid. The directional arrows demarcate the corresponding regions with clarity. (B-C) PCR analysis of *CcT1* deletion and complementation. The upstream fragment, downstream fragment, *bar*, *CcT1*, and C-*CcT1* were amplified with the primer sets up *CcT1*-F/up *CcT1*-R, dn*CcT1*-F/dn*CcT1*-R, *bar*-F/*bar*-R, *CcT1*-F（P1）/ *CcT1*-R（P2）,P3/P4, and C-*CcT1*-F/ C-*CcT1*-R, respectively (see Table S1).

Table S1 Primers used for gene disruption

| Gene | Primers Name | Sequences (5’→3’) | Notes |
| --- | --- | --- | --- |
| *CcT1* | up*CcT1*-F | TTGATATCGAATTCCTGCAGAAAGCCAACAACACCACCACCCC | For constructing knockout gene vector |
|  | up*CcT1*-R | ACGGATCCCCCGGGCTGCAGGTCGGCGCAAAATCGCA |  |
|  | dn*CcT1*-F | ATGAACTAGTTCTAGAATGTCGCAAAGTAGCTGACG |  |
|  | dn*CcT1*-R | TGGCGGCCGCTCTAGATAAAGATGACCAACCCCTGCG |  |
|  | *C-CcT1*-F | TTGATATCGAATTCCTGCAGCTTGGCCTTGTGCACATTGTC | For constructing gene complementation vector |
|  | *C-CcT1*-R | GGCCCCCTGTCGAGCTGCAGGAGAGTCTTCTCGAAGACCA |  |
|  | *CcT1*-F（P1） | CCGTGAGAGGTTTCAAGTGT | For PCR detection |
|  | *CcT1*-R（P2） | TCCCGGATACAGCCAAAGTT |  |
|  | P3 | TCAGGCTACCTACCGCTCAA |  |
|  | P4 | GGTGGTCGGCTTCTTCTTAA |  |
| *bar* | *bar*-F | TCGTCAACCACTACATCGAGAC | For Bar plasmid validation |
|  | *bar*- R | GAAGTCCAGCTGCCAGAAAC |  |
| *ben* | *ben*-F | GGTAACTCCACCGCCATCCA | For Ben plasmid validation |
|  | *ben*-R | GCAGGGTATTGCCTTTGGCACTT |  |
| *gfp* | *gfp*-F | CATCCAAGAACCTTTAATCGAATTATGGAGCAAGGCACTGCT | For PCR detection |
|  | *gfp*-R | GCTCCTCGCCCTTGCTCACCATCCGATCGAGCGCTTGACT |  |

Table S2 Gene expression analysis

| Primers Name | Sequences (5′→3′) |
| --- | --- |
| Actin-F | GGCGAGACCAGGGTACATAGTG |
| Actin-R | GCGTGAAATCGTTCGTGAC |
| phosphoglycerate mutase-F | CTGCTCGACGCTTTGCTT |
| phosphoglycerate mutase-R | CTTCCCGATTTCGCCTAC |
| Pyruvate kinase-F | CGACAGTATCGCCAGTAGTCAG |
| Pyruvate kinase-R | GCTTCGCCCACCTTTACC |
| Branched amino acid aminotransferase-F | TGAAGATGTGGCAGTAGCCG |
| Branched amino acid aminotransferase-R | CAGGACTTGAAGGTGGAGGG |
| 2-aminoadipate transaminase-F | CAAGATGGAAAACCCAAAGC |
| 2-aminoadipate transaminase-R | AGGCGAAGGGGAAGACAGT |
| Fructose diphosphate aldolase-F | GCATCCGCAACGATAAGA |
| Fructose diphosphate aldolase-R | GACAAACGGTGCTCCAAA |
| 3-deoxy-7-phosphate heptanoate synthase-F | CCGTGTCCTCCCAGTGAA |
| 3-deoxy-7-phosphate heptanoate synthase-R | CAAGGTCGCCAAGGTTGT |
| Glycine hydroxymethyltransferase-F | ATTGCCGACTCTGTTGGTG |
| Glycine hydroxymethyltransferase-R | GGGAAGACGGAGAAGTTGAT |
| chorismate mutase -F | TCAAAGTAAAGTAGCGAAGCA |
| chorismate mutase -R | TGGGACCAAATCTCAAACCT |
| Kivr-F | CCCTGGAAACTTCAAACC |
| Kivr-R | GGAAACCCTTTATTAGGACAC |
| beas1-F | GGTCCAGTCACCATCCCACG |
| beas1-R | TTGGTCGCAAAGTACGATCAGG |
| beas2-F | GAGGGCATGTCCATGTCTG |
| beas2-R | CACCGTGGTCCTTTCTTTG |
| beas3-F | TGGCCCATTTGTTCAATACCG |
| beas3-R | GGCAGAGTTGACGCCTTCCTT |

***CcT1* gene sequence(5’→3’):** ATGGAGCAAGGCACTGCTGAAAAGCCAGGCGCTGAACTGCCACCTACAGACAGCAAACAGTCGACCCCCAAAGTTGATGCCAATAGCGCTCCCCAGCGAGAACCTAAATTTCAAGACTACGTGGTATTTTCATCCCCTCTTCTCTATGATTTGAAATTTTGCACTGACACTGACGACTCATTGACTTGTCCTCGCAGCGACTATTCCACTATGCCAACAAATGGGACTTTCTGGCCTATACCGCCGGCACTGCTGCTGCCATCGGCTCTGGTATCACTATACCGCTTCTCAACATCGTCTTTGGTAAGGAGCGAACCGCTTTGAGCAGTTATACTCGCTGATACAAGCATATCCAAATGACAGGCAAATTCGCCACCAAGTTCTCCGCCTATGCTGGTACTCAAACTCTGGAAAAGGGCCAATTCCAAGGGGAGCTCAACAAGCTATCGTATGCTCTCCCTTCTCACTGCGACCGAAAACTTCCCCAATTCTTACGACTGCTAATGTGTTACTTGTGCAGCCTCTATATGCTCGGCCTTTTTCTTGGTCGCTTTGTCCTTAGCTACATCAACCAGGTGCCCATTGCTTCCTTCTATACGCTTGCTTCTGCTGACCCGATCGTAGCTCGCTTTCCGTATGACGGGCATTCGAATTTCCTCGGCCCTTCGACAAGATTTCCTCACCGCCTTGTTTTCCCAAAGCGTCCATGTACTAGACTCGATGCCTCCTGGATACGCCACCACCGTCATCACAACTGCCAGCAACACTGTTCAACTCGGAATTTCCGAGAAACTCGGTGTATTCTTCGAGTACAATGCGACAATGATTGCTTCGATTATCATTGCCTTTGTATACAGCTGGCAGCTTTCGCTGGTCACATTTACCGCAGTCGTCTTTATCGTGCTCAGCGTCAGTATCCTGCTGCCTCCCATCACGCAGGGCCAGACGCGTCAAGGCCAAGTAGGATGCCATCCACTCCACCATTGACTGTACTTATGTTAACATGAAATCAGTCTGACGCCAAGTCTGCCTCTATTGCCAGTGAAGCGCTGAGTAGCCTGAAAATGGTTGTTGCTTGCTGCGCTGAAGGCCGAATTGGCACCAAATATGGGCGATTTGTCGACGAAGCCAGAGCACATGCACAGAAAATCGGTCCCTTAATTTCAGTCCAGTTCGGCTTGATTGTATGCCAGGTTGCCCTTCCAGACAGCAACGGTGATCCACTAACATTACGATGTAAATATAGTTTTTCAGCAGTTATGCCGCCTTTGGCCTTGCATTCTGGTACGGCACCAAACTTTTGGTCGAGCAGAAGATCAATGAGCTCGGGGCTATCATCGTGTAAGTCCATCTTCCGCGCCCTGGTCCTTGTTATGCCGGGAAGGCCATGTCAGGCCGCTCTAACAACGACTTAGGGTTTTATTTTCGGTCATGATGATTGTGGCTTCAATGGAGCGAACCTCAACACCTCTTTTGGCTGTTGGAAAGGCTATGATTGCCGCTTGCGAGTTCTTTACTGTCATCGATGCGCCTAGGCCTGATCCTGGTCATCTCCGCGATCCAGATGTGTCCCCGACTGAGGATCTCGTTTTTGAGAACGTTACTTTCGCGTATCCAAGCCGACCTCATGTCAAAATTCTCGATAATCTCAACCTCACGATAGCAAAAGGCAAGGTCACTGCTTTGGTTGGGCCGTCCGGCTCTGGCAAGAGCACAATTGTCGGCCTAGTCGAACGGTGGTACAGTCTCAGCAGCCAACATATCATCTCTAAAACGATCGATACAACAAAGAAAGAGAAAGGGGACGAGAAAAACAATAAAGATGACCAACCCCTGCGAAAGAGTTCGGAAGAAGAGGACGACACTGGCCCGCCTGTCGAACTCCATGGCAGGGTGTCTACCTGCGGGCACTCTCTAGATGACATCAACATAAAATGGTGGCGGTCACAAATTGGCCTTGTTCAGCAGGAACCCTTCCTCTTCAATGATACCATTTATAAGAATGTTGTAAATGGTCTTGTTGGAACCAAGTGGGAGAACGAGCCGGAACAAAGGAAGAAGGAGATGGTTAGGGATGCTTGTAAAGAAGCATTTGCCGACGAATTTATTGAGAAACTCACGGAAGTAAGTACATAAGAACAGGATCGATAGGGCCAACGGGGATCCTCCAACATAAGGGATGCTAACCTGTCTTTATAGGGCTACGACACGCCTGTCGGCGAAGGTGGCGCGAAGCTCTCCGGTGGTCAGCGTCAACGCATTGCTATTGCCCGCGCCATCATCCGTCAACCCTCAATTCTGATTCTTGATGAAGCCACCAGCGCAATCGATGTCCGAGGCGAGAAGATTGTGCAAGCAGCCCTAGACAAGGCGTCGAAGAACAGAACCACCATTACAATAGCCCATCGCCTCTCCACCATTAAAAAGGCGGACAGGATCGTCGTTTTGAGACAGGGGCAAGTTGTCGAATCGGGTACCCACGAGAGTCTTCTCGAAGACCAAAAAGGGTTATACCACAGCCTTGTTCATGCTCAAGCACTATCCATGGAAGACGCCACAGAACCCAATCATGCCGAGGTCAGGGAAGAGCCCATATCACTTATCCGTGAGAAGAGCCGTGCCAAGTCAGAAGCAGCAGAAAATCTTCCCAAGAAGAATTACAAGACGAAGAGTATATTTTCTAGCTTCGGTCGCTTCTTTTACGAGACAAGGTCAAACTGGTGGATGATGGCTCTGGCCGTCGTCTTTTCTGCTTGTGCCGGTGCCGCCATTCCCTTTCAATCCTGGTTATTTGCCAAGGTTATCCTCGTCTTTAGTTACTTCCCGGATACAGCCAAAGTTCGACAGGAAAGTCAATTCTGGTCTCTGATGTGGACAGTCCTGGCGATTGCTGCTGGCGTCAGCTACTTTGCGACATTCTTCTTTTCCAACCGCACCGCCAGCACCATTCGCGCCAAGTATCAGAAGCAGTATTTCTCGTCAATTTTATACCAGCAATTTTCTTTTTTCGATGACGACGACCATTCTCAAGGTACCATGACTGCTCGGAGCTCGAGTGATCCACGTCAATTGGAAGAGCTTCTCGGCACGAACATGGCGAGTGTCTTCATCGCCATTTGGACGCTTACAGGTACAATCGCCATTGCCTTTGCGTTTGCTTGGAAATTGGCCCTGGTCTCGTTTTGTGTCGTCGTTCCGATTCTGCTGGGCACTGGCTATTGGCGATTCCGCTATGAACTACAGTTTGATGAAATGAACACTGCAGTGTTTGCGGACAGCTCTAAATTCGCTTCAGAAGCTATTGGAGCTTTTCGGACGGTGGTATCTCTCACGCTTGAAGATTCTATTTGCGATCGATTTAGCAACCTGGCTCGCGGCCATGTTACAGATGCATACAAGAAAGCTCGCTGGGTGACGGTTCTGTTTGCCTTTTCAGACAGCGCAACGATAGGCTGCCAAGCGCTGGTGCTGTACTATGGTGGTCGGCTTCTTCTTAACGGAGAATTCAGCCTACAGAGCTTCTTTGTCTGCTTTATTTCCGTCTTGAACGCGGGTGAAACAACAGGGCGAGCTCTGAGCTTTGGTCCCAACGTCGCCCAAGTCAGCGGAGCGGCCAACCGCATACTCAGTCTGAGGGCCAGTCAGGTGAAGGATGACCCTGCTGCCAGCGGCGAGTTGTTCAAGTCTGACGGCGACGGTATGAAGATTGAATTGGACAACATCACATTCAAGTACCCAACGCGAGATGCACCAGTTTTCAGGGGACTCAGTCTCACAATCGAAAAGGGCCAATTTGCAGCTCTCGTTGGAGCATCTGGATGTGGCAAATCGAGCATCATTTCCCTACTTGAGCGGTAGGTAGCCTGAGCTGCTCACAAATTGGAGCCATTGAGATATACTGACATCTCTCGAAACTAGATTCTACAACTTGGATGGTGGCAGAATTCTTTGCAACGGCCAGGACATTGCTGCAAACAATGTGTACGCATATCGCAGCCATCTCTCATTGGTAGCTCAAGAGTCCAGCTTGATGCAAGGTACGAGAAAAAGAACATACACCGATAATTCTTTACAAGAAACTATCAGATACCTTTACTAACGTCAATAATTTTCCGCGAAGGCACGCTACGAGAGAACATTCTACTCGGAGTCGACGAAACCACGGTGACGGACGAGATGATTCATGATGTCTGCCGCCAAGCGTCCATTCATGACTTCATCGTTTCGCTGCCCGAAGGGTACAATACGAACATTGGCTCGCGTGGCGTCTCCCTCTCCGGAGGGCAAAGGCAACGCGTAGCCATTGCGCGAGCTCTCATTCGCAACCCTGACATTTTACTACTTGATGAGGCAACCAGTGCGCTGGACTCGGAAAGCGAGAAGCAGGTGCAAGCGGCTTTTGAGCTCGCAGGAAAAGGACGCACGATGCTCGCTGTTGCGCACCGGCTTGCAACGGTGCAGAACGCGGATGTCATTTTTGTCCTTGGCGAGGGTCAGCTGCTAGAGAAGGGCAACCACCAGGAGCTTCTTGCCAAGCGAGGCGTCTACTGGCAAATGGTATGTATTTGCTTGTTGGTTTGAATTTGTCGAGTGATGCTAACTGTCGGGTTGTCCAGTGCCAGAGTCAAGCGCTCGATCGGTA
